# The Impact of New Entrepreneurial Spirit on Cultivating Entrepreneurial Values and Entrepreneurial Ability of College Students

**DOI:** 10.3389/fpsyg.2022.870455

**Published:** 2022-06-24

**Authors:** Ping Li, Xiaozhou Chen

**Affiliations:** ^1^School of Economics and Management, South China Normal University, Guangzhou, China; ^2^School of Business Administration, Guangdong University of Finance, Guangzhou, China

**Keywords:** new media, new entrepreneurial spirit, college students, entrepreneurial values, cultivation of entrepreneurial ability

## Abstract

The objectives were to deeply study the impact of new entrepreneurial spirit on the cultivation of entrepreneurial values and entrepreneurial ability of college students. First, the influencing factors of college students' entrepreneurial values were analyzed based on new media, entrepreneurial spirit, entrepreneurial values, and other related theories. Second, the corresponding questionnaire was designed and elaborated on the four aspects of college students' entrepreneurial values, namely, entrepreneurial competence, entrepreneurial risk, and entrepreneurial ethics. Finally, the data results of the questionnaire were studied. The results show that in entrepreneurial values of college students, they believe that entrepreneurship is the most important for personal development, with an overall average of 3.86. In the point of view of entrepreneurial competence, they think that independent learning ability plays a significant role in entrepreneurship, with an average of 3.91. In the view of entrepreneurial ethics, they consider that the law is the most crucial during the entrepreneurial, with an average value of 4.35. It means that college students still have certain legal knowledge. After analyzing the results of the questionnaire on entrepreneurial factors, it is found that college students lack social experience and have a low ability to take risks. Meanwhile, entertainment is the first choice for college students on new media platforms, and the time of viewing is more than 5 h/day. Therefore, the influence of new entrepreneurial spirit is researched on the cultivation of entrepreneurial values and entrepreneurial ability of college students, which provides a new theoretical basis and optimization direction for entrepreneurial education in universities in the future.

## Introduction

### Background

With the continuous development of the “Internet +” model and the emergence of the new media industry and the in-depth application of information technology, there has been a climax of innovation and entrepreneurship in college students. New media is regarded as the product of new technologies, and the latest technologies such as digitalization, multimedia, and network are all necessary conditions for the emergence of new media. After the birth of new media, the form of media communication has undergone tremendous changes, such as subway reading and large screens in office buildings, all of which have transplanted the communication content of traditional media into a new communication space. Innovation and entrepreneurship are entrepreneurial activities based on innovation, which are not only different from pure innovation but also different from pure entrepreneurship. Innovation emphasizes pioneering and originality, while entrepreneurship emphasizes the behavior of obtaining benefits through practical actions. Innovation and entrepreneurship are the trends of the times, and it is the driving force for the continuous improvement of the level of social and economic development (Wu et al., 2020). However, due to the lack of experience of college students in innovation and entrepreneurship, there are a series of problems in the process of practical operation (Yuan and Wu, 2020).

### Literature Review

Gorgievski et al. (2019) proposed that entrepreneurial values were a theory that entrepreneurs recognize entrepreneurial goals based on personal needs and entrepreneurial behavior standards. Therefore, the entrepreneurial activities of college students stem from actual entrepreneurial needs, such as survival and entrepreneurship (Gorgievski et al., 2019). Kruse et al. (2019) considered that in terms of innovation, entrepreneurship itself was an innovation process. In the booming information age, successful entrepreneurs were undoubtedly also innovators. In addition, entrepreneurial spirit can provide some guidance for the difficulties and risks encountered by college students in the process of entrepreneurship (Kruse et al., 2019). Sanchez et al. (2019) thought that entrepreneurship specifically referred to the establishment of a new enterprise and a new career exploration process. In essence, entrepreneurship was an innovation process, and entrepreneurs often have a positive view of various types of entrepreneurship (Sanchez et al., 2019). Calza et al. (2020) thought that entrepreneurship can be decomposed from the dimension of values, including entrepreneur value, national value, and social value. The main driving force of most entrepreneurs is the national value (Calza et al., 2020). Poblete (2020) pointed out that social responsibility is essential for successful entrepreneurs. Correct values were the foundation of entrepreneurship and a key competitive advantage, guiding the entrepreneurial process (Poblete, 2020). Wang et al. (2021) believed that personalized education is an innovative education method of respecting individual differences. With the progress of the times, the number of innovative and entrepreneurial talents has become one of the key indicators for measuring comprehensive national power. Therefore, while cultivating the independent thinking and problem-solving abilities of students, colleges and universities should also respect the creativity and subjectivity of students and lay a solid foundation for students' innovation and entrepreneurship in the future (Wang et al., 2021).

With the continuous development of online media, a new entrepreneurial spirit has had a huge impact on college students' entrepreneurial values and entrepreneurial ability training. The specific factors are reflected in the personal abilities and levels, the guidance of the family environment, the school's teaching of entrepreneurial knowledge, etc. (Alammari et al., 2018).

(1) Personal abilities and levels of college students
  The main factors affecting the personal abilities and levels of college students are the cost of funds, human capital, and their own psychological capital. Funds are the basic guarantee for college students to start a business. The amount of funds determines the scale of the business. Money is the blood of an entrepreneur or a start-up business. If there is no fund flow, many projects will not function properly (Goldstein et al., 2018; Matteucci, 2021). Human capital is conducive to broadening information acquisition channels of entrepreneurs, improving their ability to collect and integrate information so that it can reduce the level of information asymmetry between entrepreneurs and the market and help entrepreneurs make more correct entrepreneurial decisions. Psychological capital is a kind of positive psychological ability that can be measured and developed and help to improve work performance. It mainly reflects the positive or negative mentality held by college students in the face of difficulties and obstacles during their entrepreneurial process (Zheng et al., 2018; Raitis et al., 2021).
(2) The guidance of the family environment
  In terms of guidance of family environment, the main influencing factors are concentrated in: the influence of family background on entrepreneurship and the cultivation of family entrepreneurship. The family environment also plays a vital role in the entrepreneurial consciousness and entrepreneurial psychology of college students. The results show that if there are successful entrepreneurs in the family, it will also have a certain positive impact on others (Lechner et al., 2018; Morales et al., 2020; Ha, 2022).
(3) Teaching of entrepreneurial knowledge in school
  From the perspective of colleges, the level of entrepreneurial education depends on the requirements of teachers, the design of the goals, and so on (Fayolle et al., 2018). For college teachers, to do a good job in teaching the entrepreneurial philosophy of college students, they need to thoroughly interpret policies and treatments for entrepreneurship, have a deep understanding of entrepreneurship, and better serve the entrepreneurial education. Moreover, after college students possess certain entrepreneurial values, they need a correct methodology and world view in the process of real practice. Actually, college students will not have the situation of knowing without learning and understanding without teaching. They must use external education to implant the correct ideology into their minds and guide them to participate in various practical activities in the correct way (Watch et al., 2019; Ma, 2021; Yaroshevsky et al., 2021).

### Significance of Research

**Figure 1:**
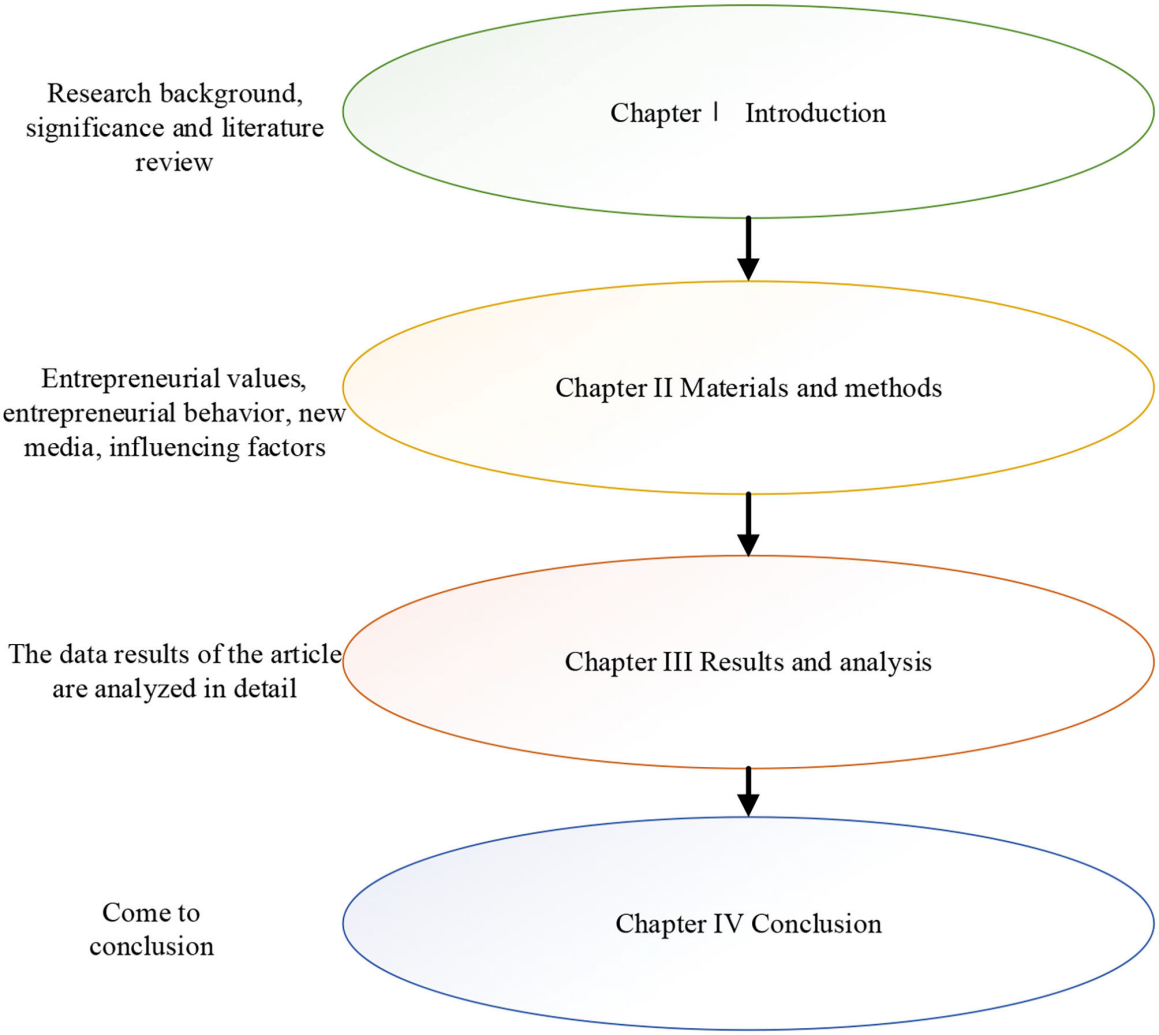
Research framework.

Based on this background, first, from the perspective of new media, college students' entrepreneurial values, entrepreneurial capabilities, entrepreneurial risk, and entrepreneurial ethics are studied. Second, a method of the questionnaire is used to investigate the results of entrepreneurial factors and the use of new media by college students. Finally, corresponding opinions and suggestions are put forward. It aims to provide a methodological reference for the future study of the entrepreneurial values of college students and the cultivation of entrepreneurial ability. The framework is shown in Figure 1.

## Model Construction of College Students' Entrepreneurial Values and Entrepreneurial Ability

### Entrepreneurial Values and Entrepreneurial Behavior

**Figure 2:**
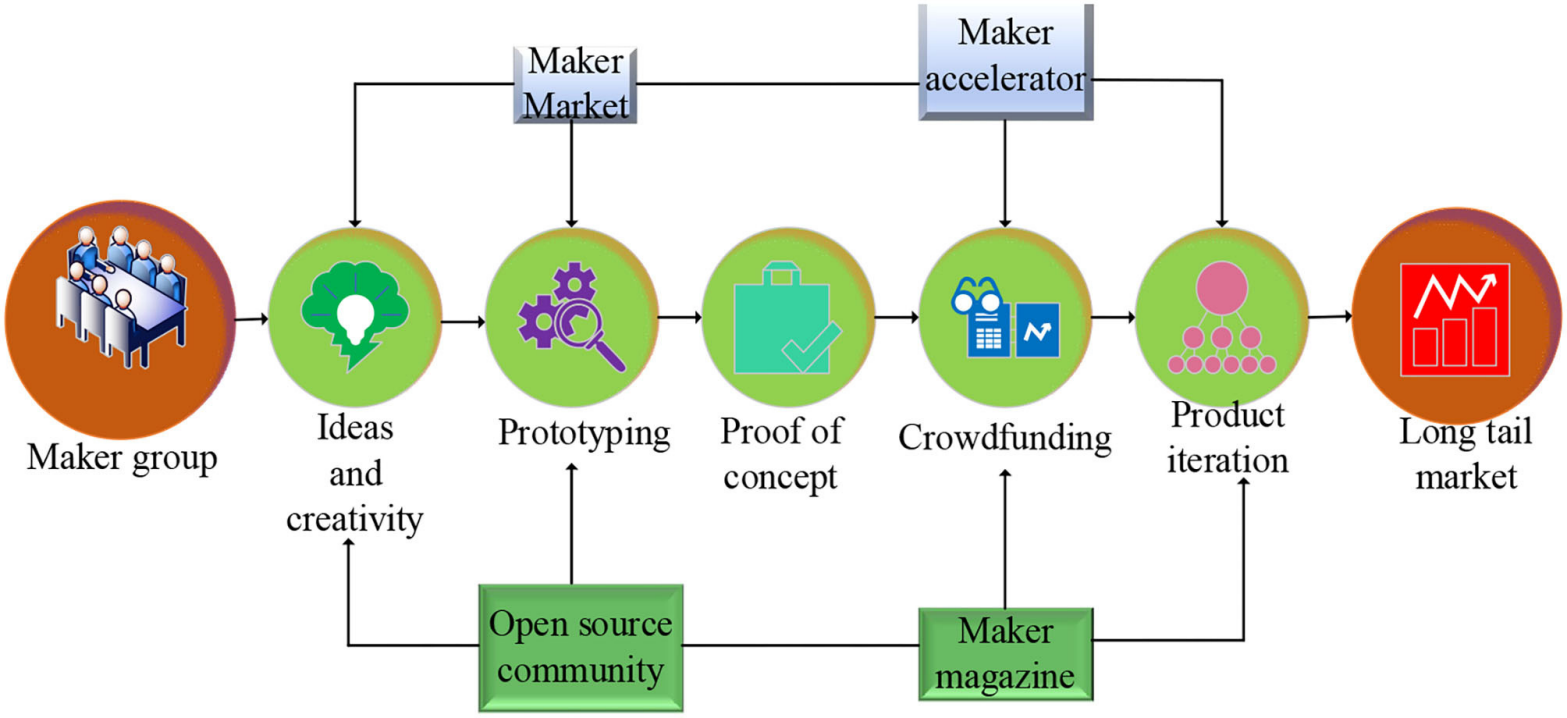
Operation mode of innovative entrepreneurship.

Innovative entrepreneurship refers to entrepreneurial activities based on one or several innovations in technological innovation, product innovation, brand innovation, service innovation, business model innovation, management innovation, organizational innovation, market innovation, channel innovation, etc. Innovation is the characteristic of innovative entrepreneurship, and entrepreneurship is the goal of innovative entrepreneurship (Siivonen et al., 2019; Watson et al., 2020). In the concept of innovative entrepreneurship, innovation is the foundation and premise of entrepreneurship, and entrepreneurship is the embodiment and extension of innovation. The fundamental difference between innovative entrepreneurship and traditional entrepreneurship lies in whether there are innovative factors in entrepreneurial activities (Dana et al., 2020; Garcon et al., 2021). The innovation in this study not only refers to technological innovation but also includes management innovation, knowledge innovation, process innovation, and market innovation (Qazi et al., 2020). In short, any activity that can bring new value to resources is innovation. Innovative entrepreneurship has the characteristics of high risk, high return, and promotion of growth (Huggins and Thompson, 2018; Oma et al., 2021). The current typical entrepreneurial process in China is expressed in Figure 2.

**Figure 3:**
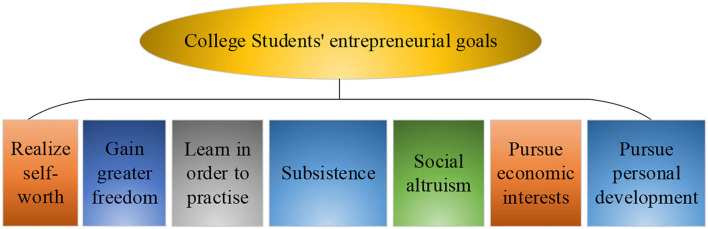
Entrepreneurship goals of college students.

Innovation ability refers to the ability to comprehensively use existing technology and knowledge, apply new methods in various activities and fields, and creatively propose new discoveries, new theories, and new programs with economic, social, and scientific value. It is a comprehensive reflection of people's ability to ask questions, analyze problems, and solve problems in the process of innovation. Innovation ability is closely related to intelligence, but intelligence is not the whole of innovation ability. A large number of experiments have proved that in addition to intelligence factors, nonintellectual factors including spirit and personality also have a great influence on people's innovation ability. The intelligence level of most people is not very different. Therefore, whether a person can achieve innovative results, nonintelligence factors will be a crucial condition. Entrepreneurial values are the meanings that the subject produces to things and behaviors in the interaction between the subject and the object. The evaluation standard of utility is the core factor that promotes the decision-making and action of the subject (Nwankwo and Gbadamosi, 2019). Values are the value criteria for judging right and wrong, true and false, good and evil, and beauty and ugliness, which are at the core of social culture. Under the guidance of entrepreneurial values, college students choose entrepreneurial projects to carry out entrepreneurial activities according to the realistic entrepreneurial conditions and resources. Entrepreneurial values can only be slowly established on the way to the actual implementation of entrepreneurship. College students need to adjust the goals, means, and evaluations of entrepreneurial value according to their own entrepreneurial values at different stages of the entrepreneurial process. (Chen, 2019). The specific content of college students' entrepreneurial goals is illustrated in Figure 3.

**Figure 4:**
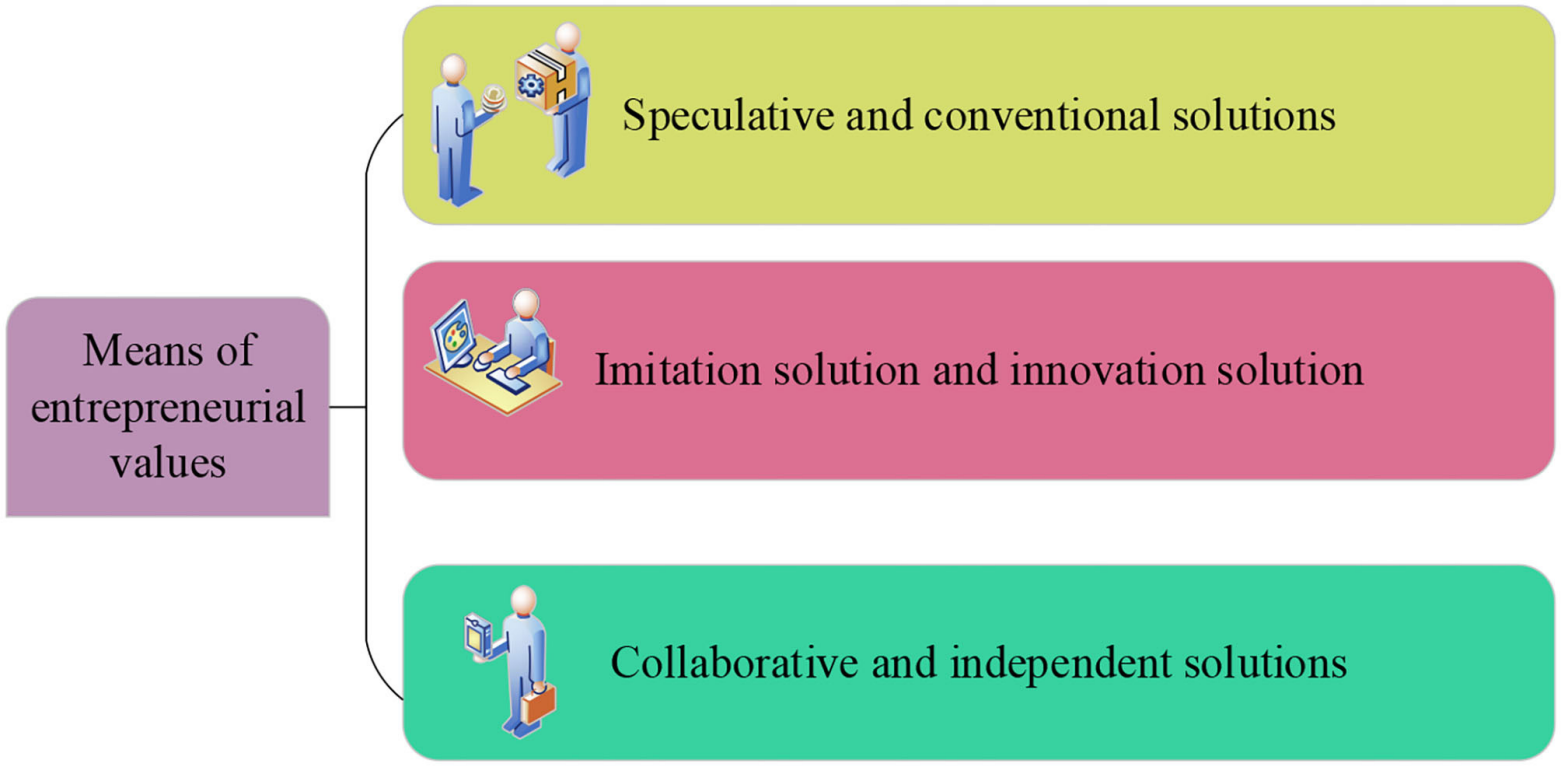
The means of entrepreneurship values of college students.

The means of the entrepreneurship value refers to the ways and means adopted by college students to achieve the goals set in advance in the process of entrepreneurship. It also refers to how entrepreneurs to start their entrepreneurship and how to achieve their entrepreneurial goals. College students will face a variety of practical problems in the process of entrepreneurship, such as changes in national entrepreneurship policies and regulations, the rapid changes in the market economy, and what entrepreneurial value methods to choose to deal with practical problems (Arkush, 2019; Darling et al., 2019). The specific means are shown in Figure 4.

### Characteristics of New Media and Entrepreneurial Spirit

**Figure 5:**
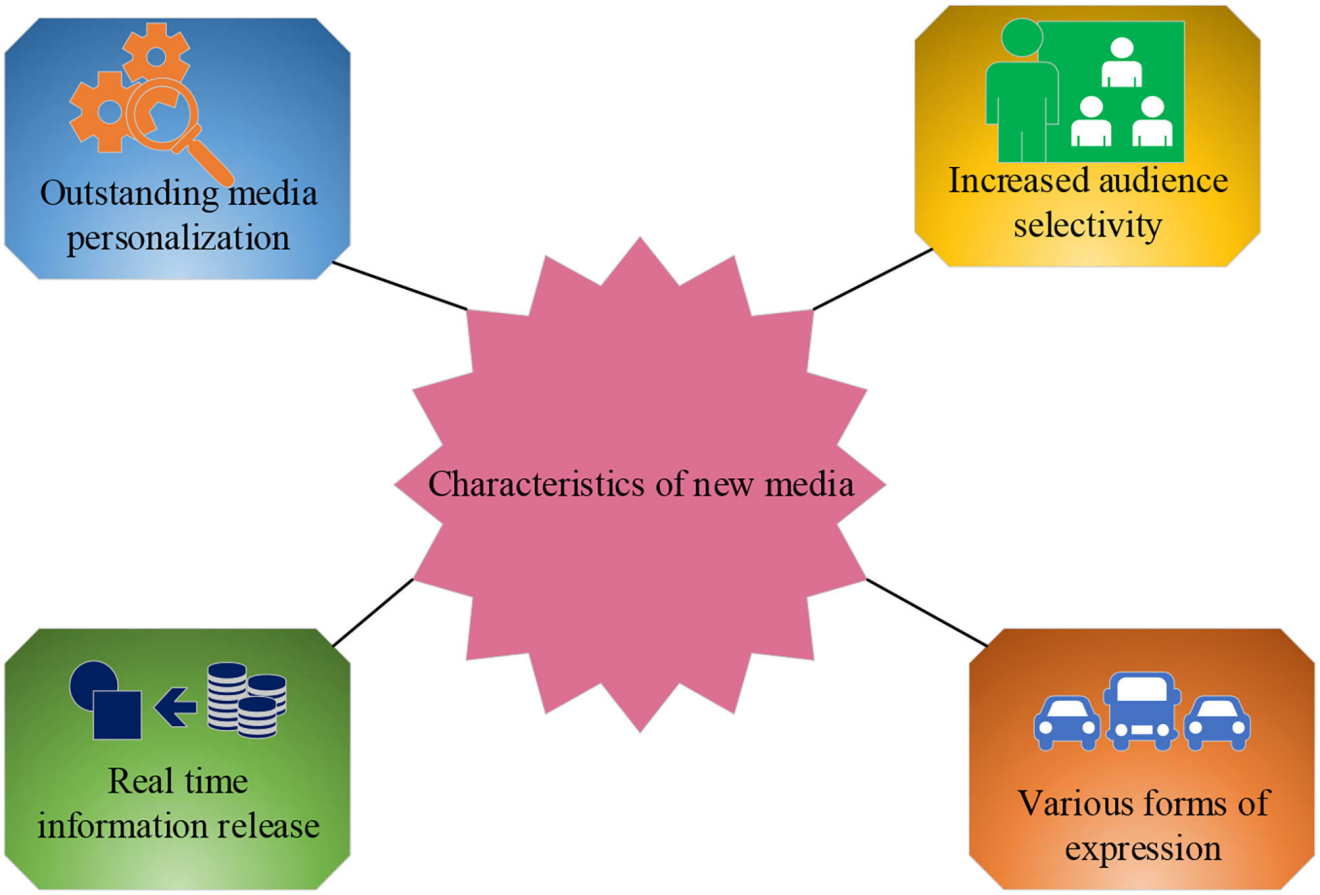
The characteristics of the new media.

With the advancement of computer and information technology, various types of new media have emerged, which have attracted more audiences through interactive changes in traditional Internet technologies and communication modes (Hueso et al., 2020). The principle is mainly to use digital technology to provide users with a form of transmission of information and services through computer networks, wireless communication networks, satellites and others, as well as computers, mobile phones, digital televisions, and other terminals (Fernandez and Romero, 2019). The characteristics of the new media are shown in Figure 5.

**Figure 6:**
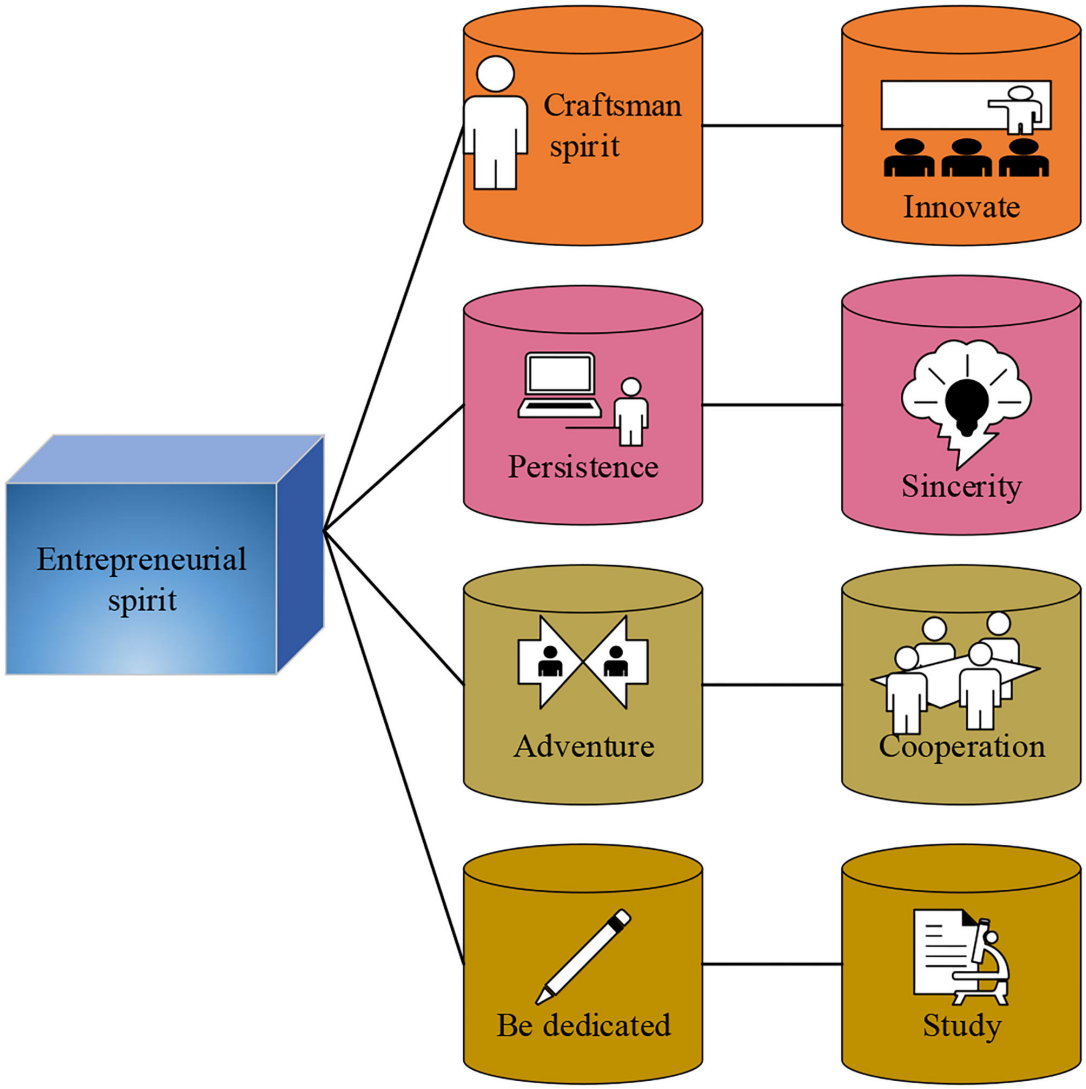
The characteristics of true entrepreneurial spirit.

Entrepreneurial spirit refers to the special ability of certain people to organize resources such as land, labor, and capital to produce goods, find new business opportunities, and develop new business models (Sang, 2022). In fact, true entrepreneurial spirit has the following characteristics. The details are shown in Figure 6.

### Design of a Questionnaire of College Students' Entrepreneurship Values

(1) Purpose of the survey

The impact of new entrepreneurial spirit on the cultivation of entrepreneurial values and entrepreneurial ability of college students is studied under the background of new media. Through the data analysis of the results, further suggestions for improving the quality of teaching strategies in colleges are put forward, to help college students increase their interest in entrepreneurship and improve the cultivation of entrepreneurial ability. Therefore, before the start of the research, the hypothesis that “new entrepreneurial spirit has a positive influence on college students' entrepreneurial values and cultivation ability” is put forward, and the conclusion will be verified in the following paragraphs.

(2) Object and standard of the survey

Students from a certain university in Sichuan Province are taken as the research object. Starting from 1 May 2019, and ending on 30 November 2019, the specific time is from 7:00 to 8:00 for students' self-study at night every Sunday. The survey standard is that 300 students from freshman to senior year in humanities and social sciences majors, economics and management majors, science and engineering majors, and sports and art majors are selected to issue questionnaires to them. The basic characteristics of the interviewed college students are displayed in Table 1.

**Table 1:** The basic characteristics of the interviewed college students.

**Basic feature**		**Number (person)**	**Percentage (%)**
Gender	Male	150	50
	Female	150	50
Discipline and specialty	Humanities and	65	21.7
	Social Sciences		
	Economic management	76	25.3
	Science and Engineering	81	27
	Sports Art	78	26
Grade	Freshman	68	22.7
	Sophomore	76	25.3
	Junior	74	24.7
	Senior	82	27.3

**Figure 7:**
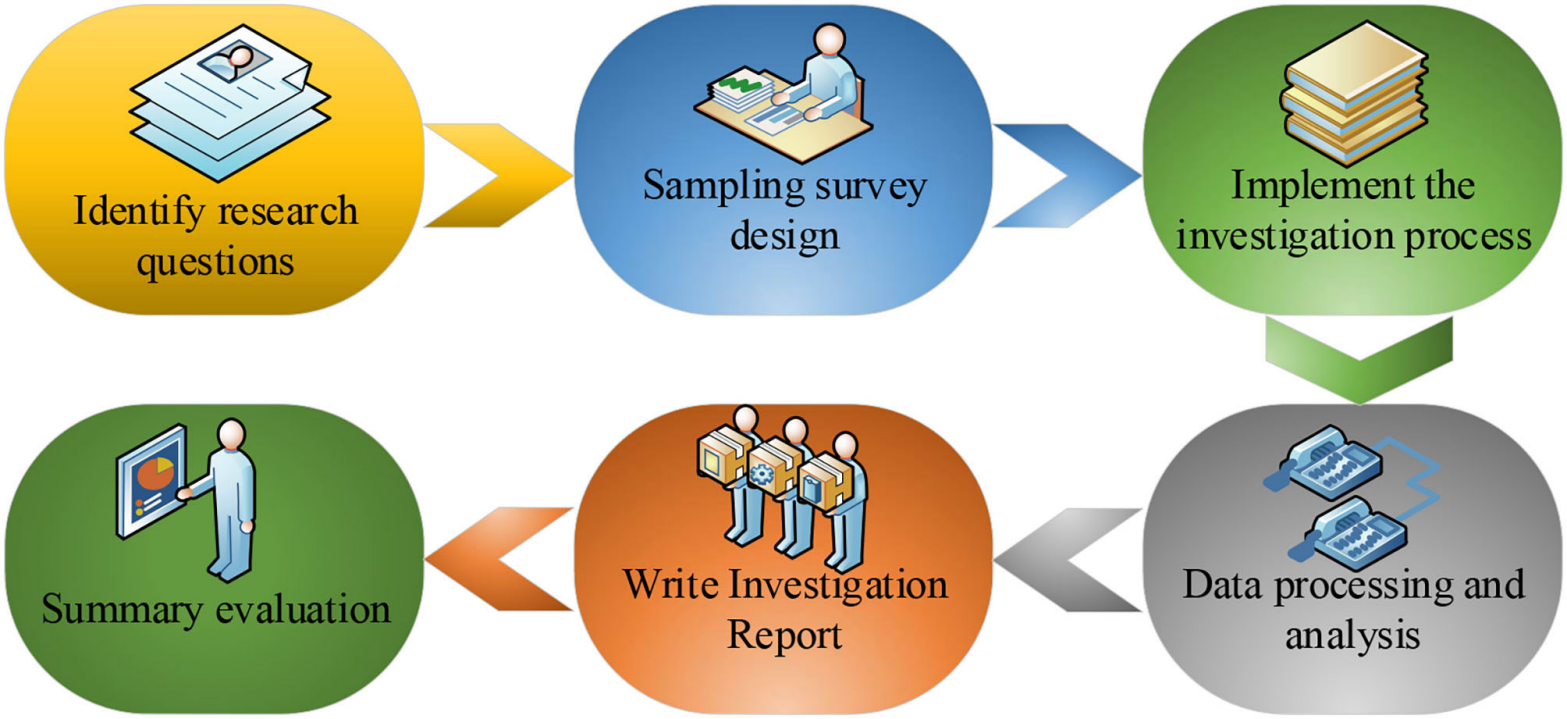
The steps of investigation in the questionnaire.

To ensure the scientificity of the questionnaire, the questionnaire is discussed with relevant professional experts before it is issued, and the unreasonable places are revised in the questionnaire. To make sure the corresponding recovery rate, the form of face-to-face distribution and on-site recovery are adopted. 263 copies are recovered, and the recovery rate is about 87.7%. The effective number of recovered copies is 241, and the effective recovery rate is 91.63%. The specific steps of investigation are exhibited in Figure 7.

To make the questionnaire results more accurate, the validity test of systematic error variance is introduced, as exhibited in the following equation:


(1) $$\begin{array}{l} r=\frac{\sum XY-\frac{\sum X\sum Y}{N}}{\sqrt{\sum X^{2}-\frac{{\left(\sum X\right)}^{2}}{N}\sqrt{\sum Y^{2}-\frac{{\left(\sum Y\right)}^{2}}{N}}}} \end{array}$$

where *r* represents the correlation coefficient, *X* denotes the dependent variable, *Y* denotes the independent variable, and *N* refers to the quantity.

According to equation (1), the fractional difference of the actual number can be obtained, as displayed in the following equation:


(2) $$\begin{array}{l} \alpha =\frac{K}{K-1}\left(1-\frac{\overset{K}{\underset{i=1}{\sum }}\sigma_{Yi}^{2}}{\sigma_{X}^{2}}\right) \end{array}$$

where α means a coefficient, *K* demonstrates a quantity, and the meanings of other letters are the same as those of equation (1). Generally speaking, the higher the reliability coefficient, the higher the reliability between variables, showing the higher the degree of internal consistency between variables. The internal consistency reliability test is carried out on the responses of the questionnaire by equation (2). The calculated result is 0.86, indicating that the reliability of the questionnaire is relatively high. The specific results are indicated in Table 2.

**Table 2:** University teachers' evaluation of the content and data of the questionnaire.

**Title**	**Number of people**	**Proportion of very satisfied (%)**	**Proportion of relatively satisfied (%)**	**Commonly (%)**	**Proportion of dissatisfaction (%)**
Professor	3	33.3	66.7	0	0
Associate professor	3	33.3	66.7	0	0
Lecturer	2	0	50	50	0
Assistant	2	50	50	0	0

Table 2 demonstrates that among the 10 teachers selected, there are 3 professors, 3 associate professors, 2 lecturers, and 2 teaching assistants. These 10 teachers maintained a very satisfied and relatively satisfied attitude. It manifests that the selected questionnaire questions and the obtained experimental data have a certain validity.

Furthermore, to test the correlation between the selected questionnaire research data, the statistical software SPSS25.0 is used for data analysis, and the specific results are demonstrated in Table 3.

**Table 3:** Correlation test results between different variables.

**Project**	**Students of different genders**	**Entrepreneurial values of different students**	**Entrepreneurial risk view of different students**
Students of different genders	0.712**	0.5856**	0.5961**
Entrepreneurial values of different students	0.5269**	0.5697**	0.6245**
Entrepreneurial risk view of different students	0.634**	0.6893**	0.6358**

Table 3 indicates that after testing the correlation between students of different genders and their values and risk attitudes toward entrepreneurship, it is found that the correlations between the values and risk attitudes of students of different genders toward entrepreneurship are 0.712, 0.5856, and 0.5961, respectively, which prove that there is a positive correlation between them. The correlations between students with different entrepreneurial values and gender and entrepreneurial risk outlook are 0.5269, 0.5697, and 0.6245, respectively, indicating that they are also positively correlated with each other. For students with different entrepreneurial risk outlooks, the correlations with gender and entrepreneurial values are 0.634, 0.6893, and 0.6358, respectively, which also indicates that they are also correlated, and the correlation is the highest among the three groups of data. Generally, 0.7 or above illustrates a very close relationship, 0.4–0.7 means a close relationship, and 0.2–0.4 stands for a general relationship. It denotes that the selected questionnaire data have a good correlation between the factors, which can meet the research requirements.

(3) Main content of the survey

**Figure 8:**
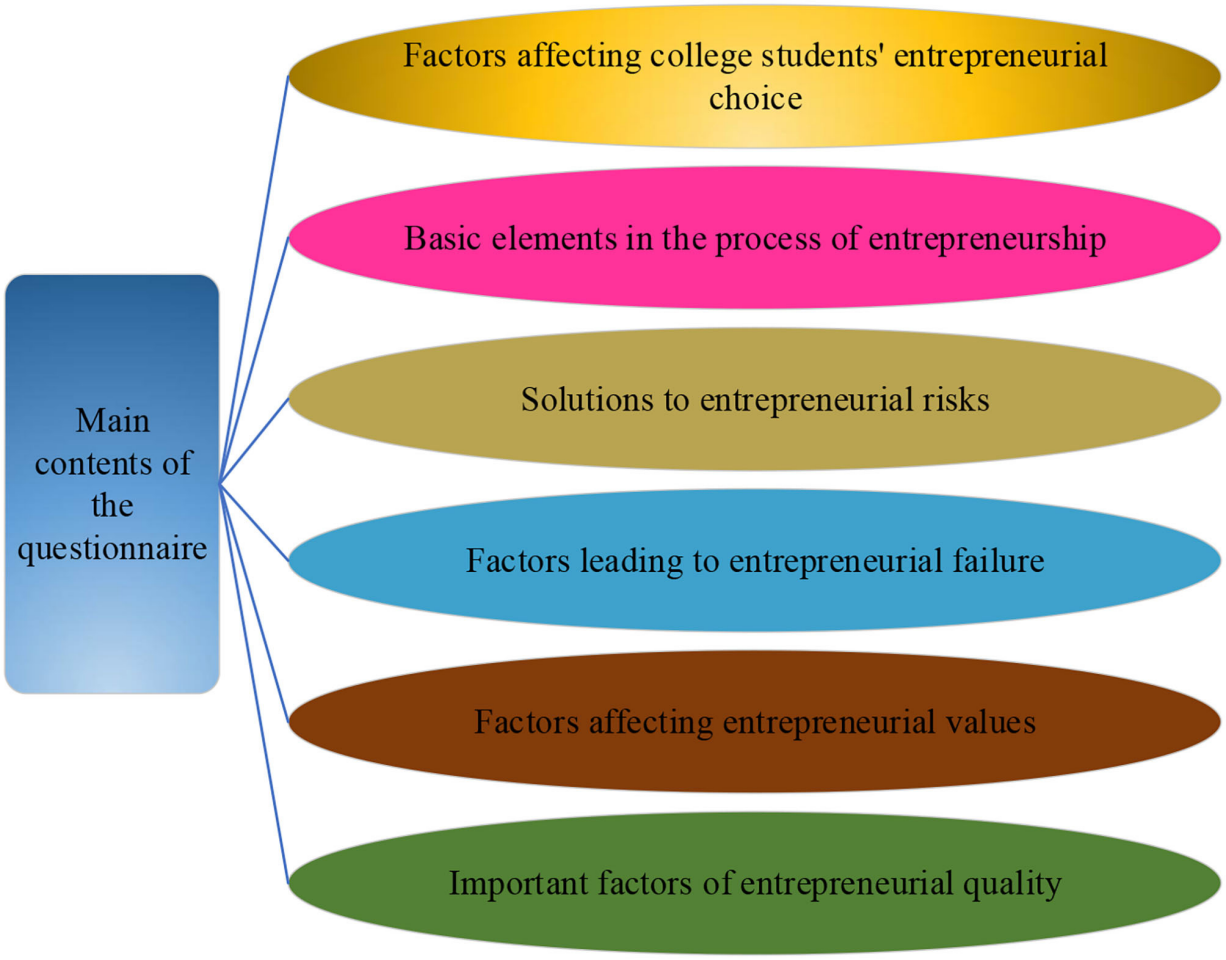
The specific content of the questionnaire.

College students are the main force of entrepreneurship. The specific content of this questionnaire is shown in Figure 8.

In Figure 8, the questionnaire survey is mainly carried out from six perspectives, which are the factors that affect the field of entrepreneurship that college students choose, the basic elements that should be possessed in the process of entrepreneurship, the solutions to face the risks of entrepreneurship, and the factors that lead to the failure of entrepreneurship, the factors that affect college students' entrepreneurship, and qualities that should be possessed before starting a business. To sum up, it is the plans and strategies before, during, and after starting a business.

## Analysis of the Results of the Cultivation of Entrepreneurial Values and Entrepreneurial Ability of College Students

### Analysis of the Results of the Questionnaire on Entrepreneurial Factors

**Figure 9:**
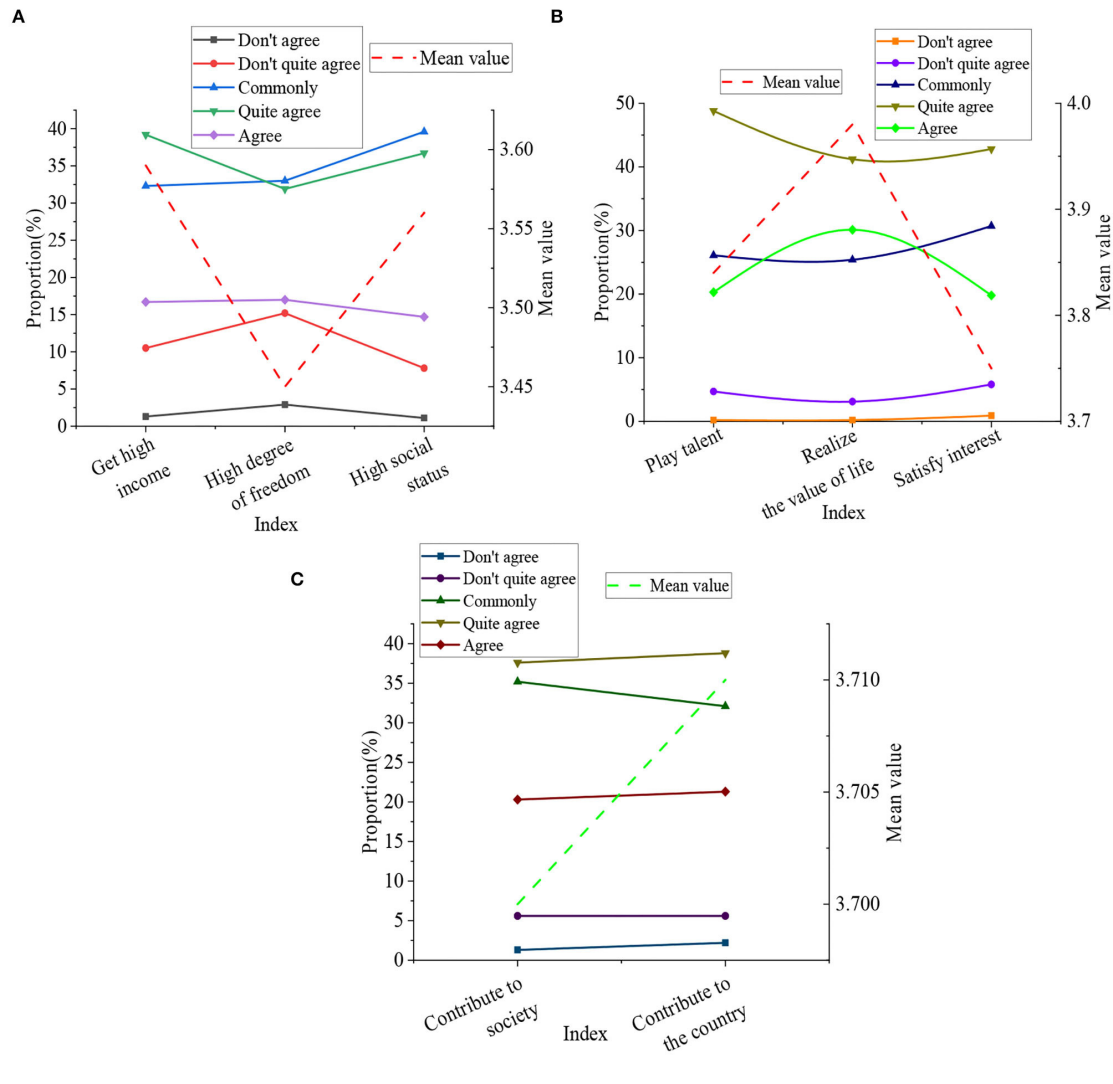
Basic situation of college students' entrepreneurship values. **(A)** shows the type of personal survival. **(B)** is the type of personal development. **(C)** means the type of social contribution.

To understand the entrepreneurial values of college students at this stage, the method of questionnaire survey was used to study it, and then the collected data were processed by SPSS 25.0. The specific results are displayed in Figure 9.

Figure 9 indicates that the entrepreneurial values of college students are divided into three types. They are personal survival, personal development, and social contribution. In the type of personal survival, the mean value of the indicator that entrepreneurship is for obtaining high income was 3.59. The average value of the indicator was 3.45 that entrepreneurship is for unconstrained, and the mean value was 3.56 that entrepreneurship is for higher social status. In the type of personal development, the mean value that entrepreneurship is to show own talents was 3.84. The mean value was 3.98 that entrepreneurship is to realize one's life value, and the average value was 3.75 that entrepreneurship is to satisfy one's hobbies. In the type of social contribution, the mean value of the indictor that entrepreneurship is to contribute to society was 3.7, and the mean value was 3.71 that entrepreneurship is to contribute to the country. It denotes that in the consciousness of most students, entrepreneurship is essential to realize the value of life. To further verify this result, respondents selected the questionnaire at that time to conduct a return visit to confirm that the results were correct. The reason why this happens is that the college students at this time have not yet entered society, and they are still full of enthusiasm for themselves and have a certain fighting spirit.

**Figure 10:**
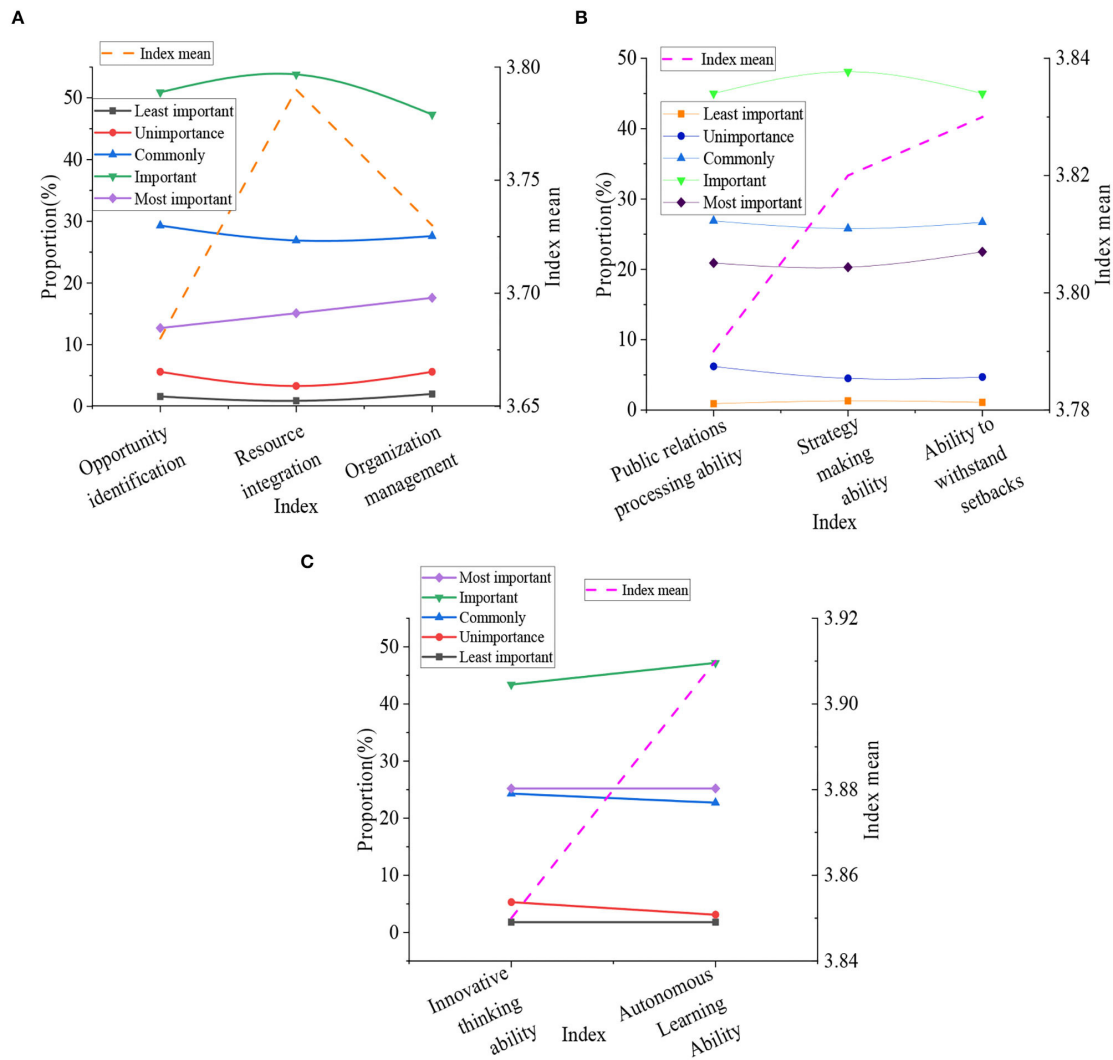
The basic situation of entrepreneurial abilities of college students **(A)** shows the recognition, integration and management ability of college students' entrepreneurship; **(B)** is the public relations, strategy formulation and frustration ability of college students; **(C)** means the thinking and autonomous learning ability of college students.

Besides, a survey is conducted on the concept of entrepreneurial abilities of college students, and the specific situation is indicated in Figure 10.

Figure 10 represents that the average value of the indicator of identification ability of entrepreneurial opportunity was 3.68. The average value of the indicator of integration and utilization ability of entrepreneurial resource was 3.79. The average index of organization and management ability of the entrepreneurial team was 3.73, and the index of public relations processing ability should be possessed in the entrepreneurial process, which was 3.79. The average indicator of strategy-making ability was 3.82. The mean value of the indicator of frustration ability was 3.83. The mean value of the indicator of the innovative thinking ability in the entrepreneurial process was 3.85, and the average value of independent learning ability was 3.91. From these data, it can be found that most students believe that the ability of autonomous learning plays an important role in entrepreneurship.

**Figure 11:**
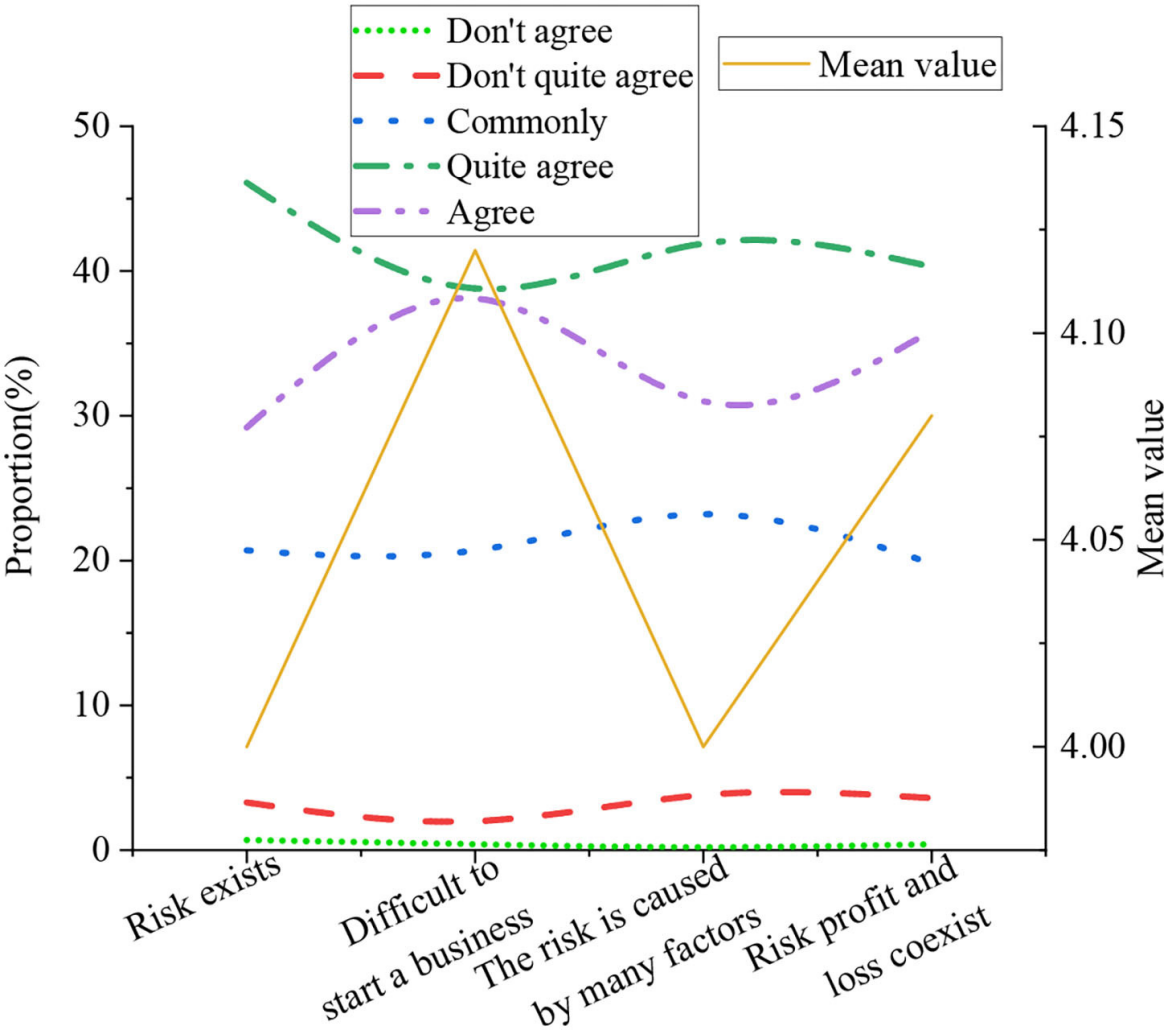
The basic situation of the entrepreneurial risk.

The basic situation of the entrepreneurial risk is illustrated in Figure 11.

Figure 11 expresses that the average value of the indicator that college students believe that entrepreneurial risk exists objectively was 4. The mean value of the indicator that entrepreneurship cannot be smooth and easy was 4.12. Entrepreneurial risk is caused by internal and external uncertain factors at the same time, and the average value of the indicator was 4. Entrepreneurial risk is an indicator of the coexistence of profit and loss, with an average value of 4.08. This shows that college students can rationally view the entrepreneurial risks that exist in the entrepreneurial process, which helps to improve the prudence of entrepreneurial decision-making of college students, and can effectively manage risks and reduce financial difficulties to a certain extent.

**Figure 12:**
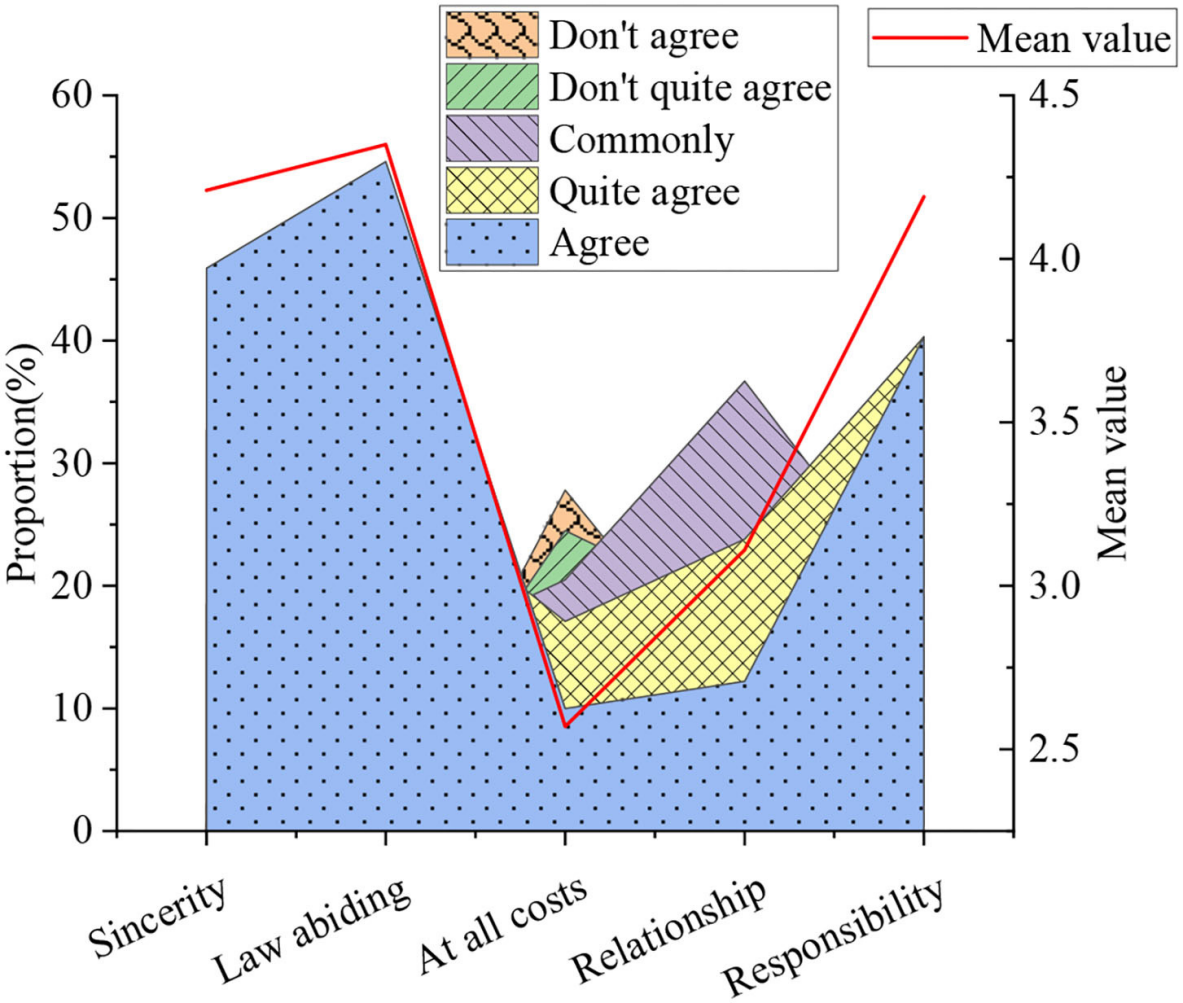
The basic situation of entrepreneurship ethics.

The basic situation of entrepreneurship ethics is demonstrated in Figure 12.

Figure 12 shows that in the process of entrepreneurship, college students believe that the average value of the indicator of integrity was 4.21, and the average value of the indicator that must have a certain legal awareness was 4.35. The average value of the indicator that can start a business at any cost was 2.57. Bribery is a necessary indicator with an average value of 3.11, and it must be realized that the average value of the index that has a certain responsibility to employees was 4.19. This indicates that college students think that the law is the most significant in the entrepreneurial process.

### Results of a Questionnaire on the Use of New Media by College Students

**Figure 13:**
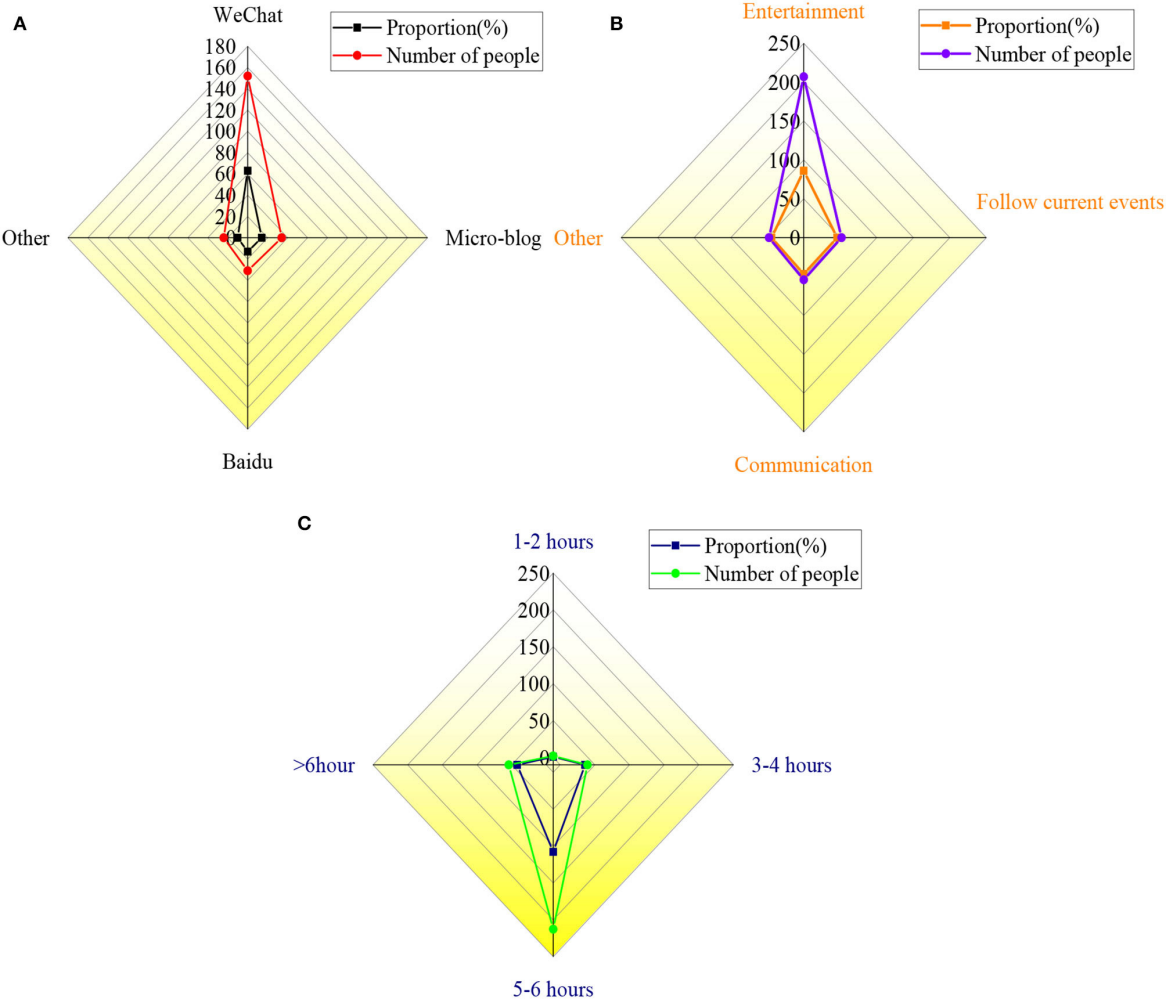
Analysis of specific results of using new media for college students **(A)** shows the types of new media used by college students; **(B)** means the purpose of college students using new media; **(C)** is the time of college students using new media.

New media is one of the indispensable tools in the process of entrepreneurship of college students. After investigating it, the specific results are displayed in Figure 13.

Figure 13 indicates that new media platforms, such as WeChat, Baidu, and Weibo, have been widely used in the entrepreneurial process of college students, with usage rates of 63, 13, and 14%, respectively. Among the three sets of data surveyed, freshmen are more inclined to use WeChat. Besides, college students use new media platforms mainly for entertainment, accounting for about 86%, for communication and communication, about 6%, and about 5% for watching current political news. The middle-aged or the elderly are more interested in real-time news and daily communication on new media platforms. In addition, the survey results denote that more than half of the respondents use Weibo, WeChat, and other software for at least 5 h/day, accounting for about 96% of the total, and about 4% for <5 h.

### Strategies of College Students' Entrepreneurship Education Under the Background of New Media

The above research shows that the current entrepreneurship education of Chinese college students is still at the stage of theoretical teaching and lacks practical capabilities to effectively cultivate students' entrepreneurial ideas and entrepreneurial ability. Some suggestions are as follows. (1) Innovative training plans need to be formulated, a complete system of entrepreneurship education needs to be built, and entrepreneurship education should be promoted. In addition, to cultivate students with a more comprehensive entrepreneurial spirit, a series of entrepreneurial management theory systems are necessary, and the teaching content should be customized for students of different grades. (2) The entrepreneurial resources of colleges should be integrated and the efficiency of entrepreneurial management should be improved. Combine a variety of factors with the cultivation of entrepreneurship education in colleges, such as the support of national entrepreneurship policies, specific guidance of enterprises, and various organizations to form a complete and effective entrepreneurial education system that integrates existing entrepreneurial resources and improves entrepreneurial management efficiency. (3) Through various college activities, entrepreneurial awareness is cultivated in an entrepreneurial atmosphere, and students are provided with reference and guidance for entrepreneurship, and a platform of entrepreneurial ability is trained. It can stimulate innovative consciousness and the entrepreneurial spirit of students in the form of social organization.

## Discussion

Strengthening the cultivation of college students' entrepreneurial values and entrepreneurial ability is one of the issues that schools, governments, and society generally pay attention to. Starting from the background of modern new media, the method of questionnaire survey is used to study how the new entrepreneurial spirit has an impact on the entrepreneurial values and entrepreneurial ability of college students. To a certain extent, entrepreneurship can bring certain inspiration and role models for college students to start their own businesses. Furthermore, college students also have their own unique understanding of entrepreneurship. Therefore, the related technologies of new media and entrepreneurship will bring positive effects to students' innovation and entrepreneurship. The highlight is that it can keep up with the pace of the development of the times and consider the trendy new media software such as Weibo and WeChat into the cultivation of college students' entrepreneurial values, which have certain timeliness. In addition, from the selection of experimental samples to the analysis of experimental results, the research results are comprehensively stated in accordance with the principle of “theory first and then methods.” However, there are still some shortcomings, such as lack of risk assessment and analysis, lack of guidance from professional innovation and entrepreneurship teachers, and so on. Therefore, in the follow-up research, the analysis of this aspect needs to be strengthened. Xu (2021) provided more suggestions to create an educational plan of “starting a business first, then graduation,” and by improving the incubation base, it encouraged lower-level college students with entrepreneurial ideas to also try the entrepreneurial practice. While teaching entrepreneurial values, taking into account extracurricular competitions, and taking advantage of the opportunities of competitions, cultivate students' in-depth understanding of production and operation, organizational financing and other links. In different colleges and universities, organizations to encourage college students to start their own businesses are formed and combined with the existing entrepreneurial resources of colleges and universities (Xu, 2021). Gatewood et al. (2020) surveyed 32 entrepreneurs from Russia and found that they have many things in common, most notably self-identity, high energy, competition, and independence. But over time, the key factors driving entrepreneurship have changed. Taking 335 entrepreneurs as a research sample, after a 6-year survey, it was found that personal perspective, goals, and self-efficacy were the main factors that directly affected entrepreneurship at the beginning, but they were replaced by mutual adjustment, tenacity, and passion in the future (Gatewood et al., 2020). The above two scholars put forward theories or methods about college students' entrepreneurship from different perspectives, but the concept of the first scholar will affect the students' course progress in the actual operation process and lack the guidance of professional innovation and entrepreneurship teachers. It will encounter many difficulties, and the operation is relatively difficult. The second scholar chose Russia as the research object to cultivate Chinese college students' entrepreneurial values, and there are certain differences. In contrast, the students of a university in Sichuan Province are directly selected as the research objects, and the experimental results are more in line with the actual situation. Furthermore, the implementation is not difficult, the economic investment is relatively small, and the operability is strong. Under the background of new media, the impact of new entrepreneurial spirit on the cultivation of entrepreneurial values and entrepreneurial ability of college students is systematically analyzed, and corresponding conclusions are drawn, which expands the practice methods of cultivating college students' entrepreneurial values and entrepreneurial ability.

## Conclusion

As young people in the new era, college students are the subjects of innovation and entrepreneurship. From the perspective of new media, the impact of new entrepreneurial spirit on the cultivation of college students' entrepreneurial values and entrepreneurial ability is studied by using the method of questionnaire survey. The main conclusions are as follows: (1) In the entrepreneurial values of college students, most students believe that entrepreneurship is the most important for individuals to realize their value in life. In the entrepreneurial ability of college students, they consider that the ability of autonomous learning plays an important role in entrepreneurship. In the entrepreneurial risk of college students, at present, they can rationally view the entrepreneurial risks that exist in the process of entrepreneurship, which helps to improve the prudence of entrepreneurial decision-making of them. In entrepreneurial ethics, college students think that law is the most significant in the process of entrepreneurship. (2) The entrepreneurial factors of college students through questionnaire, and it found that college students lack certain social experience and have a low ability to take risks. About 70% of the respondents did not show a positive attitude toward entrepreneurial risks. (3) New media platforms have been welcomed during the entrepreneurial process of college students. Most students mainly use new media platforms for entertainment, while middle-aged or the elderly are more interested in real-time news and daily communication on new media platforms. In addition, the questionnaire results show that more than 50% of the respondents use Weibo, WeChat, and other software for at least 5 h/day. Finally, through the above studies, corresponding opinions and suggestions are put forward. The innovation lies in enriching the theoretical connotation of the concept of entrepreneurship by taking the students of a university in Sichuan Province as the research object. Combined with empirical research, from the degree of college students' recognition of the current entrepreneurship education and the cultivation of entrepreneurship, countermeasures are put forward to scientifically guide college students to have a correct entrepreneurship concept. This research is guided by the relevant theories such as new entrepreneurial spirit, innovation, and entrepreneurship and constructs a related measurement index system that affects college students' entrepreneurship outlook.

There are certain limitations in data acquisition, leading to deviations in some tests of related data. The influence of new entrepreneurial spirit on the cultivation of entrepreneurial values and entrepreneurial ability of college students under the background of new media, there is no discussion on investment. The benefits evaluation can be carried out according to the specific situation in the future, so that it can provide a new theoretical basis and optimization direction for entrepreneurship education in colleges to a certain extent and help students receive better entrepreneurial education in the future.

## Data Availability Statement

The raw data supporting the conclusions of this article will be made available by the authors, without undue reservation.

## Ethics Statement

The studies involving human participants were reviewed and approved by Guangdong University of Finance Ethics Committee. The patients/participants provided their written informed consent to participate in this study. Written informed consent was obtained from the individual(s) for the publication of any potentially identifiable images or data included in this article.

## Author Contributions

All authors listed have made a substantial, direct, and intellectual contribution to the work and approved it for publication.

## Conflict of Interest

The authors declare that the research was conducted in the absence of any commercial or financial relationships that could be construed as a potential conflict of interest.

## Publisher's Note

All claims expressed in this article are solely those of the authors and do not necessarily represent those of their affiliated organizations, or those of the publisher, the editors and the reviewers. Any product that may be evaluated in this article, or claim that may be made by its manufacturer, is not guaranteed or endorsed by the publisher.
